# With Us Again

**Published:** 1863-12

**Authors:** 


### WITH US AGAIN.

Dr. A. Berry, formerly of this city, and of recent years a a resident and dental practitioner of Raymond, Miss. The Doctor found it a very warm country to live in for the last two or three years, and concluded to emigrate. Things changed so much there, during that time, that dental practice is not what it once was, nor very desirable. The Doctor has evidence of this, to the tune of about ten thousand dollars.

Upon his return, he looked somewhat as though he had passed between the upper and nether millstones.

He says he met with no very direct manifestations of hostility; except that being two or three times invited out to be hung, might be so regarded, (this may require a stretch of the imagination, however); but the Doctor objected, said he didn't want to be hung. He was very fortunate in making his escape from that land of terror and desolation. We are glad of his escape, and are much pleased that he is our neighbor. The readers of the Register will, doubtless, soon have the pleasure of hearing from him through its pages, for he wields the pen of a ready-writer.

